# Papillary cystic and solid tumour of the pancreas: Report of a case and literature review

**DOI:** 10.1186/1477-7819-3-62

**Published:** 2005-09-27

**Authors:** Abdul Kasem, Zainab Ali, Joseph Ellul

**Affiliations:** 1 Department of General Surgery, Princess Royal University Hospital,Farnborough Common, Orpington, BR6 8DN UK; 2 Department of Pathology, Princess Royal University Hospital,Farnborough Common, Orpington, BR6 8DN UK

## Abstract

**Background:**

The papillary cystic and solid tumour of the pancreas (**PCSTP**) is a rare primary neoplasm of unknown pathogenesis typically found in young women. PCSTP is a low-grade malignant tumour, which is often asymptomatic but it may present with abdominal pain.

**Case presentation:**

A 38 year old female patient who presented with one day history of epigastric pain was diagnosed as PCSTP. The patient was successfully treated with distal pancreatectomy.

**Conclusion:**

It is important to differentiate this tumour from other pancreatic tumours because, unlike malignant pancreatic tumours, this neoplasm does not usually metastasise and is amenable to cure after complete surgical resection. However, the cell origin and the aetiology of this tumour are not clear and further studies are warranted in its pathogenesis.

## Background

The Papillary cystic and solid tumour of the pancreas (PCSTP) is an unusual low-malignant epithelial tumour, which mostly affects young females with a mean age 25 years [1-3]. Nearly 90–95% of the patients are female [2-4]. It has also been referred to as a solid-cystic epithelial tumour, solid-pseudopapillary tumour or papillary-cystic tumour [5]. It makes up 0.2–2.7% of all pancreatic cancers [6,7]. Since the original description by Frantz in 1959 the incidence of PCSTP has been increasing [8], although it may be that it is increasingly being diagnosed. Furthermore, some authors believe that the tumour is not rare but occasionally is misdiagnosed as carcinoma [1]. we report here a case andpresent the literature review.

## Case presentation

A 38-year-old female presented with a one day history of acute epigastric pain. There was no nausea or vomiting. She had developed non-Insulin dependant diabetes 18 months previously. On examination she was overweight and there was epigastric tenderness, but no mass was palpable. All haematological and biochemical parameters were within normal limits.

**Figure 1:**
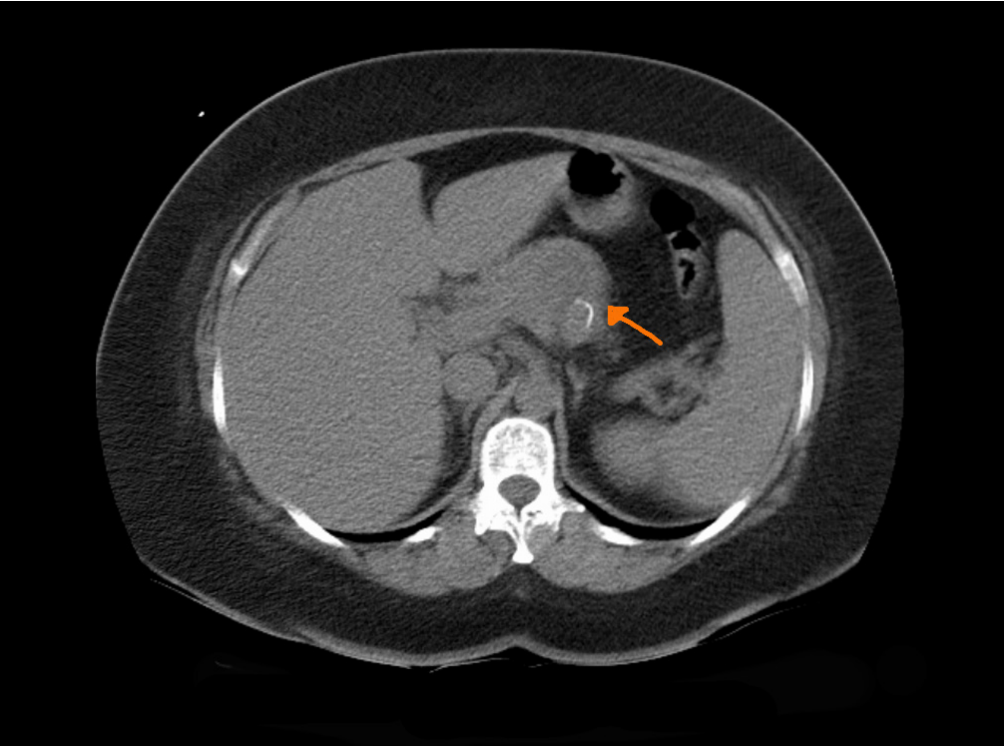
CT scan showing lesion in the pancreas.

**Figure 2:**
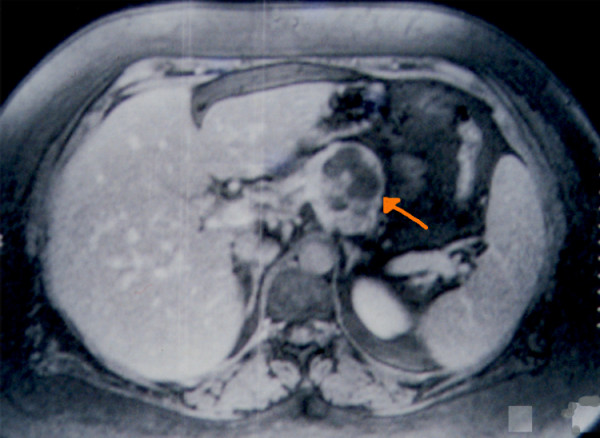
MRI showing cystic and solid lesion in the pancreas.

Abdominal ultrasonography demonstrated a 5 × 3 cm partially cystic mass in distal pancreas with a possibility of a cystic neoplasm. A contrast enhanced computed tomography (CT) scan confirmed a 2 cm mass in the body of pancreas (Figure 1). Dynamic contrast enhanced magnetic resonance imaging (MRI) showed a 4 cm cystic lesion in the body of Pancreas suggestive of a mucinous cyst adenocarcinoma (Figure 2). No liver or abdominal metastases were detected on either the ultrasound or CT examination.

The patient underwent staging laparoscopy which showed no metastases. Laparotomy was performed. An *en-block *distal pancreatectomy, including the pancreatic mass, and splenectomy was performed with full clearance of peri-pancreatic and coeliac nodes. The patient made an uneventful recovery.

**Figure 3:**
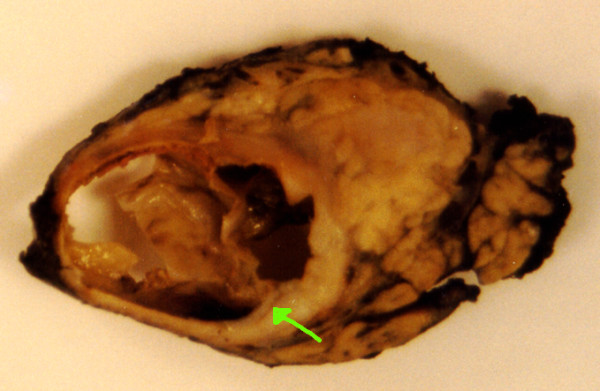
Gross specimen of the lesion.

**Figure 4:**
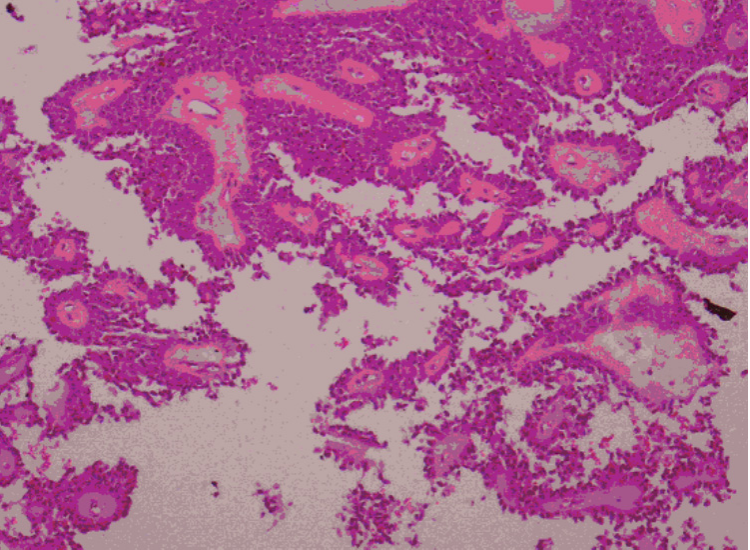
Photomicrograph of papillary cystic and solid tumour of the pancreas (haematoxylin and eosin ×40).

On gross examination the pancreatic tumour was oval, 7.0 cm in diameter, and was surrounded by a fibrous pseudocapsule. Its cut surface showed solid and cystic spaces (Figure 3). On microscopy, the solid portion of the tumour revealed sheets of uniform polygonal cells as well as dyscohesive papillae arranged around fine fibrovascular cores. The cyst wall was composed of dense acellular fibrous tissue within which the tumour cells were arranged as cords and trabeculae set within a mucinous background (Figure 4).

No invasion of the tumour into surrounding normal tissue was present. The tumour cells were strongly positive for vimentin and alpha-1 antitrypsin and showed faint focal positivity with MNF116 and chromogranin.

## Discussion

The presenting features of PCSTP are relatively non-specific. In the case of large tumours, patients present with symptoms related to compression of adjacent structures. The tumours are either found incidentally or they generally cause mild abdominal symptoms such as abdominal discomfort or chronic and acute pain. Jaundice is rare, even in tumours that originate from the head of the pancreas and usually there is no associated functional endocrine syndrome [9-12]. Rarely, these tumours are found due to haemoperitoneum from rupture of the tumour [3,13]. Physical examination might show a palpable mass and epigastric tenderness [3].

PCSTP usually arises in the tail, the body, or occasionally in the head of the pancreas. The body and the tail of the pancreas are most frequently affected [3,4]. PCSTP may appear to be extra-pancreatic with only localised connection to the gland [9]. Development of the tumour in ectopic sites of pancreatic tissue has also been described [1].

Laboratory investigations provide little additional information [14]. Elevated serum tumour markers (CEA, CA19-9) have not been described with PCSTP [8,15].

Ultrasonographic findings are not characteristic and they may be suggestive of papillary tumour [16]. In PCSTP, ultrasonography reveals a sharply demarcated, well circumscribed, variably solid and cystic mass without any internal septation [3,17]. CT scaning presents a variable picture depending on the relationship of cystic-necrotic to solid components. Typically, on CT scan PCSTP tumours appear as sharply circumscribed, well-encapsulated, heterogeneous and hypodense lesions [4,12].

Koito *et al *[18], reported that endoscopic ultrasonography may provide an accurate diagnosis of pancreatic cystic tumours<2 cm. Additional studies are warranted in this area [19].

MRI offers good visualization of haemorrhagic areas [20]. On MRI, PCSTPs are sharply demarcated and have areas of high signal intensity corresponding to foci of haemorrhage [9].

The origin and histogenesis of this tumour is controversial [3,21] and little is known about it [22]. The fact that PCSTP tumours may express epithelial as well as mesenchymal markers and occasionally show exocrine and endocrine features suggesting an origin from a stem cell. However, the nature of the tumour cells is not obvious from their structure and behaviour. In addition, their strongly sex-linked occurrence is not in keeping with an origin from a stem cell. In one case, the tumour cells were highly reactive to progesterone antibody, while they were negative for estrogen. These findings suggest a ductal origin for PCSTP, and also suggested that the sex hormones play a role in its growth, but not its genesis [23].

The neoplasm is generally encapsulated and is well demarcated from the remaining pancreas[15]. The cut surface reveals lobulated, light brown solid areas admixed with zone of haemorrhage and necrosis as well as cystic spaces filled with necrotic debris[22].

PCSTP has mixed histological features including: solid monomorphous pattern with variable sclerosis, a pseudopapillary, trabecular and microcystic patterns [24]. Although criteria of malignancy have not been clearly established [15], unequivocal perineural invasion or angioinvasion, with or without deep invasion into the surrounding tissue, is taken to indicate malignant behaviour [11,22]. The most useful markers are alpha-1-antitrypsin, alpha-1-antichymotrypsin, neuron spesific enolase (NSE) and Vimentin [22,25].

One study found a definite increase in both oestrogen and progesterone receptors in the PCSTP tumour relative to both presently determined and previously published levels in normal pancreas. The very high levels of progesterone receptor detected in this tumour support the hypothesis that the PCSTP is oestrogen-responsive, since expression of progesterone receptor is induced by oestrogen, and thus constitutes an index of effective oestrogen action in a given tissue [26]. Oestrogen and progesterone receptors have been demonstrated by biochemical assays in four solid-pseudopapillary tumour [11,26]. Immunohistologically, some studies fail to detect nuclear oestrogen receptors [11,25] while progesterone receptors had been demonstrated in eight tumours [11].

Although a few case reports suggested that the preoperative diagnosis of PCSTP is possible by using fine needle aspiration (FNA), especially in clinically typical examples [9,20,22]. FNA was avoided in many cases because of the potential risk of tumour spillage and this may compromise surgical cure [1,27]. However, cytology obtained via FNA may not be useful to differentiate between pancreatoblastoma and PCST and if the tumour is operable, FNA is not necessary.

The differential diagnosis of PCSTP includes any cystic and/or solid pancreatic process such as: congenital pancreatic cysts, haemorrhagic pseudocyst, parasitic hydatid cyst, and other common cystic neoplasms of the pancreas, such as serous cystadenoma or cystadenocarcinoma, mucinous cystic neoplasms, cystic islet cell tumours, pancreatoblastoma, or mucinous duct ectasia [2,4,19]. Cases of PCSTP can mimic pancreatic cyst, occasionally mistakenly treated by cystogastrotomy [27].

Complete resection is the treatment of choice [14], and the standard therapy should involve complete removal of the tumour, the associated lymph nodes, the involved pancreas and any adjacent involved organs. Local invasion, recurrence, or limited metastases should not be considered contraindications to resection [1,27]. Surgery should be performed even if local infiltration is present, and in selected cases when complete excision is not possible, excision combined with postoperative radiotherapy to the residual mass has been used. [3]

Surgical management has been tailored to the slow-growing, non-invasive nature of this tumour. Depending on the location of the PCSTP, the surgical operation is chosen. With tumour involvement of the head of pancreas, a pylorus-preserving pancreaticoduodenectomy is recommended [27]. PCSTPs involving the neck or body of the pancreas were resected by central pancreatectomy and reimplantation of the pancreatic remnant into the stomach, with theoretical benefit of preserving pancreatic parenchyma and spleen [27]. When the tumour was located at the pancreatic tail, tail and body, or body of pancreas, distal pancreatectomy with splenectomy was employed in many cases [3]. Many authors recommended splenic conservation following distal pancreatectomy when possible [1,27].

Given the low grade malignancy and the excellent prognosis of PCSTP, conservative resection such as enucleation, evolution, lumpectomy, central pancreatectomy and partial resection of the head of pancreas have been suggested as safe and effective surgical procedures, especially in paediatric patients [3,5,16,28,29].

However, if at all possible, complete and radical excision should be the aim, as surgical curability is high and there is no clearly established role for radio-chemotherapy or embolisation in the treatment of PCSTP [20]. However, sporadic reports are present. Matusuda *et al*, reported a case of multiple hepatic metastases which responded to chemo-embolisation of the tumour [1], Fried *et al*, observed substantial shrinkage of an unresctable tumour after 6 weeks of radiotherapy [1].

PCSTP is a remarkably indolent neoplasm, and is regarded as a carcinoma of low malignant potential [9]. Most authors consider PCSTP as a benign or low-grade malignancy [20]. More than 95% of patients with PCSTP limited to the pancreas are cured by complete surgical excision. Clear resection margins are necessary to prevent local recurrence [9]. Surprisingly, even patients with metastatic disease have experienced long-term survival[9].

## Conclusion

PCSTP is uncommon primary pancreatic neoplasm of unknown aetiology with low malignant potential generally occurring in young women. The sex and age distribution suggests that hormonal factors may be important in the pathogenesis of PCSTP. PCSTP should be considered in the differential diagnosis of any pancreatic mass, especially in young women. Unlike other pancreatic malignant tumours, this neoplasm is indolent and metastases are rare. The treatment of choice is complete surgical removal and the prognosis is excellent after complete surgical resection.

## Competing interests

The author(s) declare that they have no competing interests.

## Authors' contributions

**AK**: Reviewed the literature and prepared the draft manuscript

**ZA**: Contributed the photomicrographs and pathological part of the manuscript

**JE**: Supervised the manuscript preparation and edited the manuscript

All authors read and approved the manuscript
